# GETTING BETTER OR GETTING WORSE: MARKERS OF LONGEVITY ACROSS COHORTS AND COUNTRIES

**DOI:** 10.1093/geroni/igad104.1336

**Published:** 2023-12-21

**Authors:** Peter Martin, Yasuyuki Gondo, Oscar Ribeiro

## Abstract

The expansion of life expectancy and the lengthening of the human life span are extraordinary achievements observed over the last century. Among those accomplishments is the increasing number of centenarians in many countries. However, it is not clear whether new cohorts of centenarians are in better health when compared to earlier born cohorts of centenarians. The purpose of this symposium is to compare centenarian cohorts across different countries. Two centenarian cohorts each from Hong Kong, Japan, and the United States are included in the analyses. Results provide different perspectives across countries. The Hong Kong Centenarian Studies compared current centenarians to those who participated at an earlier time and noted that proxies in the second study rated centenarians in worse health when compared to centenarian self-ratings. The Kyotango study assessed living conditions, care need level, ADL, IADL, and cognitive function by gender and noted cognitive improvement for men. Finally, later cohorts of the U.S. Health and Retirement Study were in worse health when compared to the earlier born cohort. The results provide an indication for policy makers and practitioners who will prepare for a larger number of centenarians in need of additional support. Although some improvement for later born cohorts is noticeable, it is obvious that more care provision will also be needed as centenarians around the world will survive with more health conditions. Additional research should evaluate centenarians in other parts of the world.

